# Relationship between Lifestyle and Health Factors and Severe Lower Urinary Tract Symptoms (LUTS) in 106,435 Middle-Aged and Older Australian Men: Population-Based Study

**DOI:** 10.1371/journal.pone.0109278

**Published:** 2014-10-15

**Authors:** David P. Smith, Marianne F. Weber, Kay Soga, Rosemary J. Korda, Gabriella Tikellis, Manish I. Patel, Mark S. Clements, Terry Dwyer, Isabel K. Latz, Emily Banks

**Affiliations:** 1 Cancer Research Division, Cancer Council NSW, Sydney, New South Wales, Australia; 2 Griffith Health Institute, Griffith University, Gold Coast, Queensland, Australia; 3 School of Public Health, Sydney University, Sydney, New South Wales, Australia; 4 National Centre for Epidemiology and Population Health, Australian National University, Canberra, Australian Capital Territory, Australia; 5 Murdoch Childrens Research Institute, Melbourne, Victoria, Australia; 6 Karolinska Institute, Stockholm, Sweden; 7 Fielding School of Public Health, University of California Los Angeles, Los Angeles, California, United States of America; 8 The Sax Institute, Sydney, New South Wales, Australia; University of Louisville, United States of America

## Abstract

**Background:**

Despite growing interest in prevention of lower urinary tract symptoms (LUTS) through better understanding of modifiable risk factors, large-scale population-based evidence is limited.

**Objective:**

To describe risk factors associated with severe LUTS in the 45 and Up Study, a large cohort study.

**Design, Setting, and Participants:**

A cross-sectional analysis of questionnaire data from 106,435 men aged ≥45 years, living in New South Wales, Australia.

**Outcome Measures and Statistical Analysis:**

LUTS were measured by a modified version of the International Prostate Symptom Score (m-IPSS). The strength of association between severe LUTS and socio-demographic, lifestyle and health-related factors was estimated, using logistic regression to calculate odds ratios, adjusted for a range of confounding factors.

**Results:**

Overall, 18.3% reported moderate, and 3.6% severe, LUTS. Severe LUTS were more common among men reporting previous prostate cancer (7.6%), total prostatectomy (4.9%) or having part of the prostate removed (8.2%). After excluding men with prostate cancer or prostate surgery, the prevalence of moderate-severe LUTS in the cohort (n = 95,089) ranged from 10.6% to 35.4% for ages 45–49 to ≥80; the age-related increase was steeper for storage than voiding symptoms. The adjusted odds of severe LUTS decreased with increasing education (tertiary qualification versus no school certificate, odds ratio (OR = 0.78 (0.68–0.89))) and increasing physical activity (high versus low, OR = 0.83 (0.76–0.91)). Odds were elevated among current smokers versus never-smokers (OR = 1.64 (1.43–1.88)), obese versus healthy-weight men (OR = 1.27 (1.14–1.41)) and for comorbid conditions (e.g., heart disease versus no heart disease, OR = 1.36 (1.24–1.49)), and particularly for severe versus no physical functional limitation (OR = 5.17 (4.51–5.93)).

**Conclusions:**

LUTS was associated with a number of factors, including modifiable risk factors, suggesting potential targets for prevention.

## Introduction

Lower urinary tract symptoms (LUTS) represent a cluster of chronic urinary problems, generally arising as the result of disorders of the bladder, bladder neck, prostate or urethra; with LUTS most commonly attributed to benign prostatic hyperplasia (BPH). LUTS are associated with diminished quality of life [1], [2] and if left untreated, can progress to urinary retention, urinary tract infections and renal insufficiency [3].

The development and widespread acceptance of the International Prostate Symptom Score (IPSS) has increased the comparability of population based studies of LUTS in men worldwide [4]. The prevalence of moderate-severe symptoms, as measured by the IPSS, is approximately 20% among men aged over 40 years [5], [6], [7]. LUTS can be further classified into storage, voiding or postmicturition symptoms.

Much of the research to date has focussed on surgical or medical management of symptoms but there is growing interest in identifying preventive measures for reducing the burden of LUTS by identifying risk factors associated with these symptoms, especially those that are potentially modifiable [8]. Age is the primary risk factor for LUTS, with prevalence, number of symptoms and severity of symptoms all increasing with age [9]. Other risk factors include comorbidities such as diabetes, cardiovascular disease, hypertension and the side effects of the pharmacological treatments for these comorbidities [10]. Other postulated but not yet clearly established factors associated with LUTS include higher body mass index (BMI), lower socio-economic status, being married, family history, dietary and lifestyle factors (such as alcohol, caffeine, smoking, physical inactivity), history of sexually transmitted diseases, other prostate-related conditions and ethnicity [8], [11], [12], [13], [14].

The objective of this study was to examine the association between demographic, lifestyle and health-related factors that may be associated with severe LUTS, including storage and voiding symptoms separately, in men aged 45 years or older. This was achieved through analysis of data obtained from men participating in a large population-based Australian cohort study; the 45 and Up Study.

## Methods

### Study sample

The Sax Institute's 45 and Up Study is a population-based cohort study of people aged 45 and over in New South Wales (NSW), the most populous state of Australia [15]. The cohort was established with the aim of providing reliable evidence to inform health policy to support Australia's healthy ageing population. Participants were randomly sampled from the Medicare Australia database, Australia's universal health insurance system, which includes all citizens and permanent residents of Australia, some temporary residents and refugees. People aged 80 years and over and residents of regional areas were oversampled by a factor of two. Participants completed a mailed self-administered questionnaire and consent form for long term follow-up, distributed from 2006–2008. The participation rate was 18%; however, the 45 and Up study sample has excellent heterogeneity and is reasonably representative of the NSW population, comprising approximately 10% of the total population aged 45 and over. The 45 and Up Study, has a response rate comparable to similar contemporary studies internationally and in Australia, and has been shown to yield reliable data on risk factor – outcome relationships [16]. This paper uses the baseline cross-sectional data from the 123,751 men who entered the Study during the time period from 2006 to 2009. Men with missing data for age (n = 3) and those with missing data on any individual component of the m-IPSS (n = 17,313; 14.0%) were excluded leaving a sample of 106,435 men. There were more missing m-IPSS data in older age groups than younger age groups.

### Assessment of Lower Urinary Tract Symptoms (LUTS)

A modified version of the IPSS was used in the 45 and Up Study. The IPSS captures seven urinary symptoms by enquiring about men's self-reported urinary function and symptoms over the past month [4]. Three of these seven are ‘storage’ symptoms: difficulty postponing urination (‘urgency’), having to urinate again less than 2 hours after finishing previous urination (‘frequency’), having to urinate frequently during the night (‘nocturia’); and four of the seven are ‘voiding’ symptoms: having to push or strain to start urination (‘straining’), a weak urinary stream (‘weak stream’), stopping and starting again several times during urination (‘intermittency’), and the feeling of incomplete emptying of the bladder after urination (‘incomplete emptying’). The modified IPSS (m-IPSS) had identical questions regarding symptoms to the IPSS, but had a 4-point response scale (“not at all”, “sometimes”, “often”, “almost always”) compared with a 6-point response scale used on the original IPSS. For the nocturia item, respondents were asked ‘over the past month, how many times did you usually get up from bed to urinate during the night?’ The respondents chose one of the two answers, ‘never’ and ‘some nights,’ and then entered a ‘number of times per night___’ if applicable. The modification was made to ensure the entire baseline questionnaire would fit on five printed pages.

### Overall LUTS score

To allow comparability between the m-IPSS and the IPSS and to categorise men's symptoms as mild, moderate or severe, we calibrated the m-IPSS used in the 45 and Up Study with IPSS results from a representative Australian study of male urinary symptoms, MATeS [17]. The calibration procedure has been described previously [14]. Briefly, we compared frequency distributions for the summary scores of the original IPSS from MATeS and the m-IPSS from the 45 and Up Study for participants aged ≥45 in age-specific subgroups. Using standard cut-offs for the IPSS (0–7, 8–19, 20–35 for no/mild, moderate and severe symptoms, respectively) we ascertained the proportion of men in each of these categories both overall and within 5-year age groups. Cut-off points were set for the m-IPSS to ensure that similar proportions of men fell into each symptom category. Based on the calibration method outlined above, the cut-off values for clinical ranges of the m-IPSS were: 0–5 (no/mild symptoms), 6–11 (moderate symptoms) and 12–21 (severe symptoms). The main outcome used in the analyses was a dichotomous variable, with men classified as having severe LUTS (severe symptoms) or not (no/mild or moderate symptoms).

### Storage and voiding symptoms

To assess the presence of storage (irritative) and voiding (obstructive) symptoms, participants who answered ‘not at all’, ‘sometimes’, ‘often’ or ‘almost always' for a particular symptom received a score of 0, 1, 2 or 3, respectively. For nocturia, individuals who reported that they urinated 0, 1, 2 or 3 times during the night received a score of 0, 1, 2 or 3, respectively. Respondents who reported that they urinated more than 3 times per night also received a score of 3. In addition, individuals who did not report frequency but indicated they got up to urinate during the night also received a score of 1. The sum of the m-IPSS scores resulted in a range of 0–9 for storage symptoms and 0–12 for voiding symptoms. The cut-off values for m-IPSS storage and voiding symptoms were: 0–5 (low symptom score) and ≥6 (high symptom score).

### Demographic, lifestyle, and health-related variables

Demographic and lifestyle variables investigated included age, education, net annual household income from all sources, health insurance status, alcohol consumption, smoking, physical activity (measured by the Active Australia Survey [18]) and body mass index (BMI [19]). Health-related issues were determined using the question: ‘Has a doctor ever told you that you have…’ ‘prostate cancer’, ‘an enlarged prostate’, ‘heart disease’, ‘stroke’, ‘diabetes,’ ‘high blood pressure’ or ‘blood clot (thrombosis).’ Individuals were also asked ‘have you ever had any of the following operations?’ ‘vasectomy’, ‘part of prostate removed’, or ‘whole prostate removed’ and whether they had ever had a prostate specific antigen (PSA) test. Erectile dysfunction was classified based on participants’ answer to the question ‘how often are you able to get and keep an erection that is firm enough for satisfactory sexual activity?’ Male infertility was determined by the question, ‘have you ever tried for more than 1 year but have been unable to father children?’ Physical functional limitation was assessed by the Medical Outcomes Study – Physical Functioning scale (MOS-PF) [20]. Participants were also asked ‘do you regularly need help with daily tasks because of long-term illness or disability?’ Self-rated quality of life health was measured using a five-point Likert scale with response categories of ‘excellent’, ‘very good’, ‘good’, ‘fair’ or ‘poor’. Psychological distress was measured using the Kessler Psychological Distress Scale (K10) [21].

### Statistical analysis

We first calculated the prevalence of severe overall LUTS (m-IPSS ≥12) by factors related to prostate disease by 10-year age groups. Among men without a history of prostate cancer, prostatectomy or part of their prostate removed, we also calculated the proportion of men with severe LUTS by whether they had an enlarged prostate and a history of PSA testing.

For subsequent analyses we excluded men who reported ever having prostate cancer, a prostatectomy or part of their prostate removed because these factors are known to affect LUTS. We calculated the proportion of men with severe LUTS by demographic, lifestyle and health-related factors. We estimated the strength of association between these factors and severe LUTS, using logistic regression to calculate odds ratios (OR) and 95% confidence intervals (CI), first adjusting for age only, then also adjusting for education, income, alcohol consumption, smoking, BMI and physical activity. Variables used for ORs and confidence intervals included separate categories for missing values to prevent any loss of observations. ORs and confidence intervals did not change materially when the same models did not include the missing cases (results not shown). Adjusted ORs for each risk factor were also estimated for high storage symptom scores and high voiding symptom scores, separately.

Sensitivity analyses were undertaken using a different cut-point for severe LUTS in order to investigate the effect of an alternate grouping of symptoms on results. For this, we repeated the analyses for severe LUTS, classifying individuals with a m-IPSS score of ≥11 as severely symptomatic as opposed to a score of ≥12. Sensitivity analysis showed little material difference in results.

All analyses were conducted with Stata Version 12.0.

### Ethics statement

The study was approved by the University of New South Wales Human Research Ethics Committee Application (HREC Application number 05035). All respondents provided informed written consent.

## Results

Overall, 18.3% of the 106,435 men in the cohort reported symptoms consistent with moderate, and 3.6% severe, overall LUTS. Severe LUTS were more common among men reporting previous prostate cancer (7.6%), total prostatectomy (4.9%) or removal of part of the prostate (8.2%) compared with other men (**Table 1**). Prevalence of severe LUTS was even higher among men without a history of prostate cancer or prostatectomy who reported a previous doctor-diagnosis of an enlarged prostate (14.3%). Of the 95,089 men with no history of prostate cancer or prostatectomy, 79.5% had either no or mild LUTS, 17.4% had moderate LUTS and 3.1% had severe LUTS (**Table 2**). The proportion with moderate-severe LUTS increased with age, from 10.6% among men aged 45–49 to 35.4% for men aged ≥80.

**Table 1:** Prevalence of severe Lower Urinary Tract Symptoms LUTS (m-IPSS ≥12) according to doctor-diagnosed prostate health factors in the 45 and Up Study.

	Proportion with severe LUTS overall (m-IPSS ≥12)					
	% (n with severe LUTS/total)					
	Age 45–49	Age 50–59	Age 60–69	Age 70–79	Age ≥80	Total**
**Prostate cancer**						
Yes	12.5% (4/32)	6.9% (39/567)	6.9% (130/1,880)	7.2% (156/2,178)	9.4% (136/1,455)	7.6% (465/6,112)
No	1.1% (135/12,831)	2.1% (729/34,389)	4.0% (1,160/29,349)	5.1% (794/15,558)	6.1% (496/8,196)	3.3% (3,314/100,323)
**Total prostatectomy**						
Yes	6.9% (2/29)	5.0% (21/422)	3.6% (44/1,234)	4.7% (53/1,121)	7.6% (53/698)	4.9% (173/3,504)
No	1.1% (137/12,834)	2.2% (747/34,534)	4.2% (1,246/29,995)	5.4% (897/16,615)	6.5% (579/8,953)	3.5% (3,606/102,931)
**Partial prostatectomy**						
Yes	9.7% (3/31)	8.8% (26/294)	8.8% (115/1,311)	7.3% (154/2,103)	8.5% (157/1,843)	8.2% (455/5,582)
No	1.1% (136/12,832)	2.1% (742/34,662)	3.9% (1,175/29,918)	5.1% (796/15,633)	6.1% (475/7,808)	3.3% (3,324/100,853)
**Enlarged prostate** *						
Yes	13.7% (39/285)	13.9% (268/1,925)	14.8% (546/3,697)	14.0% (358/2,552)	14.4% (204/1,421)	14.3% (1,415/9,880)
No	0.8% (94/12,513)	1.4% (437/32,177)	2.1% (517/24,428)	2.7% (304/11,084)	3.4% (170/5,007)	1.8% (1,522/85,209)
**Ever had a PSA test** *						
Yes	1.9% (96/5,128)	2.5% (550/22,213)	4.1% (904/22,172)	5.3% (561/10,594)	6.7% (284/4,247)	3.7% (2,395/64,354)
No	0.5% (37/7,460)	1.2% (139/11,264)	2.4% (129/5,357)	3.2% (85/2,645)	3.9% (73/1,890)	1.6% (463/28,616)

*Excluding men with prostate cancer and/or previous prostate surgery.

**Total numbers in Table 1 do not include missing cases. Percentage missing: Ever had a PSA test 2.2%; other variables  = 0 missing.

**Table 2:** Overall Lower Urinary Tract Symptoms (LUTS) prevalence by age in the 45 and Up Study based on the modified IPSS.*

	Age in years
	% (n)					
LUTS	45–49	50–59	60–69	70–79	≥80	Total
**No/Mild (0–5)**	89.4% (11,440)	84.8% (28,928)	76.6% (21,548)	70.2% (9,567)	64.6% (4,151)	79.5% (75,634)
**Moderate (6–11)**	9.6% (1,225)	13.1% (4,469)	19.6% (5,514)	25.0% (3,407)	29.6% (1,903)	17.4% (16,518)
**Severe (12–21)**	1.0% (133)	2.1% (705)	3.8% (1,063)	4.9% (662)	5.8% (374)	3.1% (2,937)
**Mean score [SD]**	2.58 [2.44]	3.04 [2.86]	3.83 [3.31]	4.41 [3.49]	4.85 [3.66]	3.53 [3.18]
**Total n**	12,798	34,102	28,125	13,636	6,428	95,089

*Excluding men with prostate cancer and/or previous prostate surgery.

**Figure 1:**
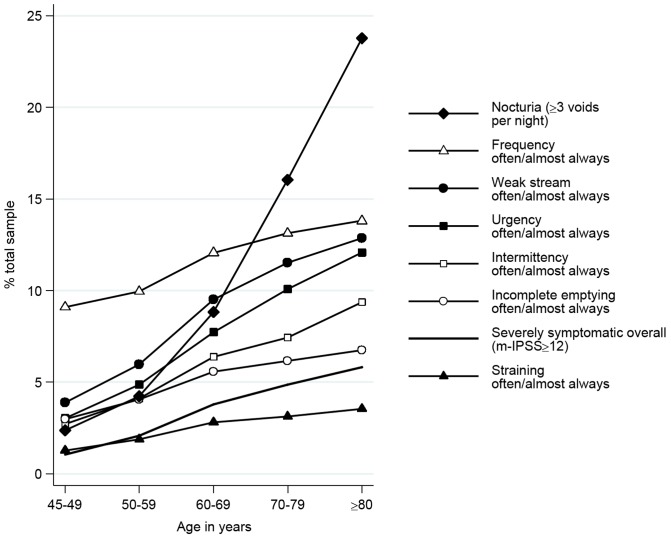
Prevalence of Lower Urinary Tract Symptoms (LUTS) by age group among men aged 45 and over.* Figure 1 displays the prevalence of each Lower Urinary Tract Symptom (LUTS) as it increased with each increase in age group (Fig 1). We excluded all men with prostate cancer or previous prostate surgery from this figure to demonstrate the effect of ageing on LUTS. Most notably nocturia (needing to void 3 or more times per night) increased from 2% in men aged 45–49 to 24% in men aged 80 years and over. *Excluding men with prostate cancer or previous prostate surgery.

**Figure 2:**
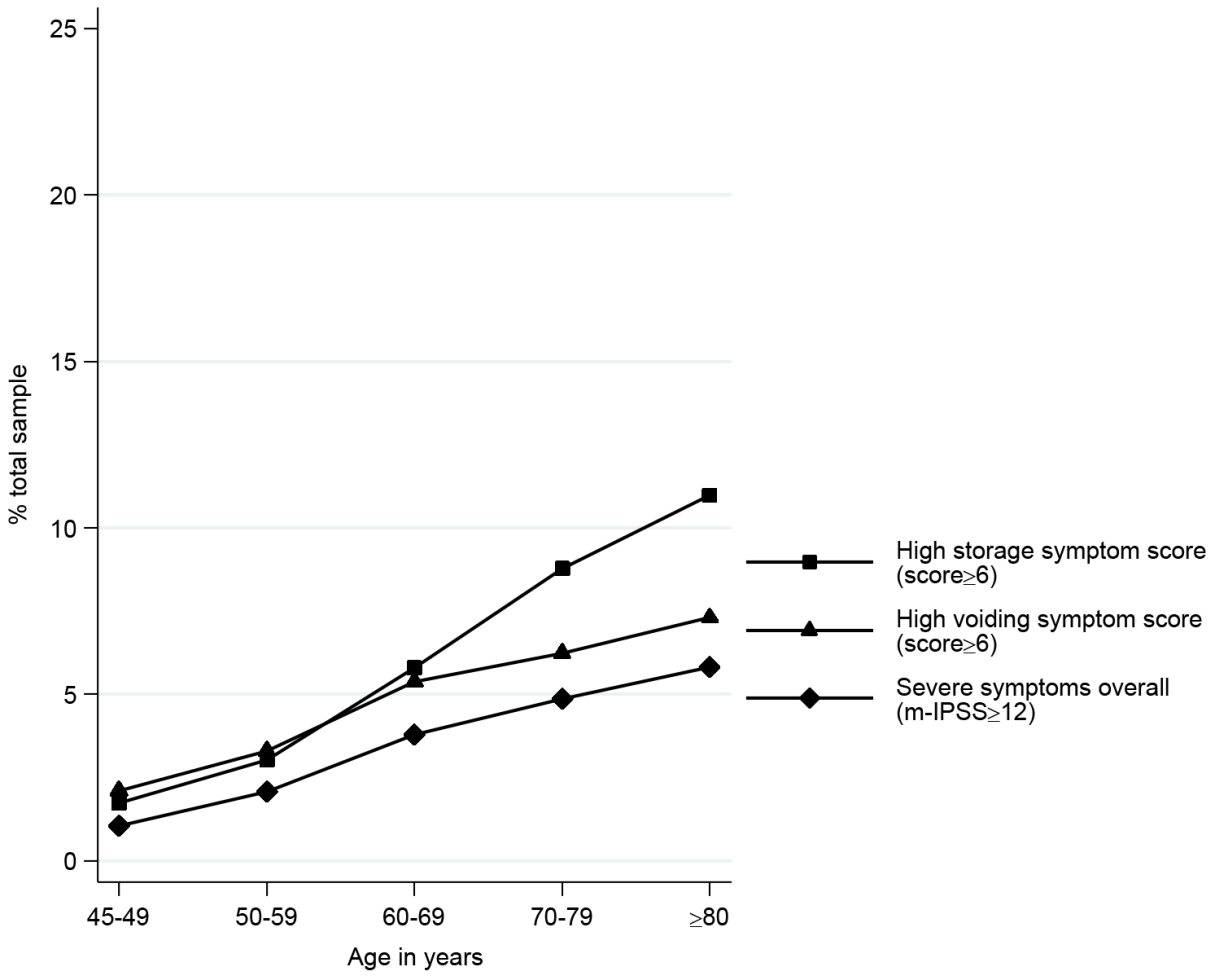
Proportion of men aged 45 and over with severe overall LUTS and high storage and voiding symptom scores, by age.* Figure 2 displays the prevalence of high storage symptom scores (scoring 6 or more on the modified International Prostate Symptom Score (m-IPSS)), high voiding symptom scores (m-IPSS of 6 or more), and high overall LUTS scores (m-IPSS of 12 or more) as it increased with each increase in age group (Fig 2). We excluded all men with prostate cancer or previous prostate surgery from this figure to demonstrate the effect of ageing on LUTS. High storage problems were more common than voiding symptoms at or above 60 years old. *Excluding men with prostate cancer or previous prostate surgery.

The prevalence of severe overall, storage and voiding symptoms all increased with age, with a marked increase in high storage symptom scores from the age of 70 (Figures 1 and 2). Nocturia was the most common symptom reported, with almost a quarter of men ≥80 years reporting ≥3 voids/night. Of the 2,937 men with severe overall LUTS, 2,653 (90%) had high voiding symptom scores; 2,222 (76%) had high storage symptom scores; and 1,938 (66%) had both high voiding and high storage symptom scores.

After adjustment for demographic and lifestyle factors, the odds of severe overall, storage and voiding symptoms increased with age; the odds of severe overall LUTS were more than 4-fold higher among men aged ≥85 than those aged 45–49 (**Table 3**). The adjusted OR for severe overall, storage and voiding symptoms were lower for men with higher educational attainment, higher income and with private health insurance.

**Table 3:** Relationship of overall, storage and voiding severe Lower Urinary Tract Symptoms (LUTS) to demographic factors and health behaviours among men in the 45 and Up Study.*

Characteristic	Total**	Severe symptoms overall (m-IPSS ≥12)			High storage symptom score (score ≥6)	High voiding symptom score (score ≥6)
		% (n)	OR	OR (95%CI)***	OR (95%CI)***	OR (95%CI)***
			adjusted for age only	full adjusted model		
**Age in years**						
45–49	12,798	1.0% (133)	1.00	1.00	1.00	1.00
50–54	16,348	1.7% (272)	1.61	1.54 (1.25–1.91)	1.42 (1.20–1.67)	1.24 (1.06–1.45)
55–59	17,754	2.4% (433)	2.38	2.22 (1.83–2.72)	1.95 (1.66–2.28)	1.84 (1.60–2.13)
60–64	15,611	3.4% (533)	3.37	2.87 (2.36–3.50)	2.79 (2.39–3.25)	2.28 (1.97–2.63)
65–69	12,514	4.2% (530)	4.21	3.24 (2.66–3.96)	3.20 (2.73–3.74)	2.45 (2.11–2.84)
70–74	8,175	4.8% (390)	4.77	3.54 (2.88–4.36)	4.08 (3.47–4.80)	2.61 (2.23–3.06)
75–79	5,461	5.0% (272)	4.99	3.56 (2.85–4.44)	4.69 (3.96–5.56)	2.45 (2.06–2.92)
80–84	4,896	5.6% (272)	5.60	3.92 (3.14–4.90)	5.17 (4.36–6.14)	2.75 (2.32–3.28)
≥85	1,532	6.7% (102)	6.79	4.76 (3.61–6.28)	6.51 (5.26–8.04)	3.17 (2.51–3.99)
**Education attainment**						
No school certificate	9,063	5.6% (509)	1.00	1.00	1.00	1.00
School certificate	13,829	3.5% (478)	0.64	0.72 (0.63–0.82)	0.79 (0.71–0.89)	0.81 (0.72–0.91)
Higher school certificate/trade	26,886	3.2% (853)	0.64	0.75 (0.66–0.84)	0.82 (0.75–0.90)	0.83 (0.75–0.92)
Certificate/diploma	18,620	2.5% (468)	0.52	0.66 (0.58–0.76)	0.76 (0.68–0.85)	0.76 (0.68–0.85)
Tertiary qualification	25,432	2.3% (583)	0.51	0.78 (0.68–0.89)	0.87 (0.78–0.97)	0.87 (0.77–0.97)
**Annual household income**						
<$20,000	15,129	6.0% (901)	1.00	1.00	1.00	1.00
$20,000–$39,999	16,188	3.7% (601)	0.64	0.72 (0.65–0.81)	0.82 (0.76–0.90)	0.81 (0.74–0.90)
$40,000–$69,999	19,434	2.5% (493)	0.52	0.62 (0.55–0.70)	0.68 (0.62–0.75)	0.72 (0.65–0.80)
≥$70,000	30,224	1.5% (447)	0.35	0.44 (0.39–0.51)	0.52 (0.47–0.58)	0.61 (0.55–0.69)
**Private health insurance**						
No	31,280	4.3% (1,347)	1.00	1.00	1.00	1.00
Yes (Hospital/DVA)	63,809	2.5% (1,590)	0.60	0.82 (0.75–0.89)	0.85 (0.79–0.91)	0.87 (0.81–0.93)
**Alcoholic drinks per week**						
0 drink	20,770	4.3% (895)	1.33	1.20 (1.08–1.33)	1.27 (1.17–1.38)	1.15 (1.06–1.25)
>0 to <7 drinks	25,609	3.0% (770)	1.00	1.00	1.00	1.00
7 to <15 drinks	23,968	2.6% (619)	0.80	0.83 (0.75–0.93)	0.92 (0.85–1.01)	0.86 (0.78–0.94)
≥15 drinks	22,583	2.6% (602)	0.84	0.79 (0.71–0.88)	0.97 (0.88–1.06)	0.77 (0.70–0.84)
**Tobacco smoking**						
Never	45,717	2.5% (1,123)	1.00	1.00	1.00	1.00
Past	41,216	3.6% (1,490)	1.30	1.31 (1.21–1.43)	1.14(1.07–1.22)	1.30 (1.22–1.40)
Current	7,853	3.9% (306)	1.88	1.64 (1.43–1.88)	1.42 (1.26–1.59)	1.46 (1.30–1.64)
**Body mass index**						
Underweight	529	5.3% (28)	1.65	1.36 (0.92–2.01)	1.27 (0.92–1.76)	1.16 (0.80–1.67)
(15 to <18.5 kg/m^2^)						
Normal weight	26,510	3.0% (794)	1.00	1.00	1.00	1.00
(18.5 to <25 kg/m^2^)						
Overweight	42,512	2.8% (1,198)	1.01	1.02 (0.93–1.12)	0.99 (0.92–1.06)	1.04 (0.96–1.12)
(25 to <30 kg/m^2^)						
Obese	19,935	3.7% (727)	1.40	1.27 (1.14–1.41)	1.41 (1.30–1.54)	1.14 (1.04–1.25)
(30 to 50 kg/m^2^)						
**Sessions of physical activity per week**						
First tertile (Low)	27,959	3.6% (1,017)	1.00	1.00	1.00	1.00
Second tertile	32,880	2.9% (955)	0.78	0.85 (0.77–0.93)	0.89 (0.83–0.96)	0.80 (0.74–0.86)
Third tertile (High)	32,248	2.8% (893)	0.76	0.83 (0.76–0.91)	0.88 (0.81–0.95)	0.77 (0.71–0.83)

*Excluding men with prostate cancer and/or previous prostate surgery.

**Total numbers in Table 3 do not include missing cases, but odds ratios and confidence intervals include the missing cases. Percentage missing: Education attainment  = 1.3%; Annual household income  = 14.8%; Alcoholic drinks per week  = 1.2%; Tobacco smoking  = 0.3%; Body mass index  = 5.9%; Sessions of physical activity per week  = 2.1%; other variables  = 0 missing.

***Adjusted for age, education, income, alcohol consumption, smoking, BMI and physical activity.

Men who reported higher versus lower levels of physical activity had lower odds of severe LUTS. Both current and past smokers had higher odds of severe LUTS than never smokers, and obese men were more likely to report severe LUTS than those with a normal BMI of 18.5–24.9 kg/m2. A similar pattern of ORs was evident for both high storage and voiding symptom sub-types (**Table 3**).

Men who reported ever being told by their doctor that they had heart disease, stroke, diabetes, high blood pressure or any cardiovascular disease were more likely to have severe LUTS than men without these conditions, with ORs all at or in excess of 1.20 (**Table 4**). ORs for both high voiding and storage symptom scores were also raised in men with these conditions. The odds for severe LUTS were almost four times higher in men with severe versus no erectile dysfunction. Odds of severe LUTS were also higher in men who reported difficulty fathering children, but there was no association between vasectomy and severe LUTS, with an upper confidence limit of 1.14. Men who required help with daily tasks had almost three times the odds of severe LUTS than men who did not, and there was a marked increase in severe LUTS with increasing physical functional limitation, increasing psychological distress and, decreasing quality of life, with a more than 8-fold increase in the odds of LUTS in those reporting poor versus excellent quality of life.

**Table 4:** Risk of severe overall, storage and voiding Lower Urinary Tract Symptoms (LUTS) according to self-reported health measures among men in the 45 and Up Study.*

Characteristic	Total**	Severe symptoms overall (m-IPSS ≥12)			High storage symptom score (score ≥6)	High voiding symptom score (score ≥6)
		% (n)	OR	OR (95%CI)***	OR (95%CI)***	OR (95%CI)***
			adjusted for age only	full adjusted model		
**Heart disease**						
No	81,658	2.7% (2,234)	1.00	1.00	1.00	1.00
Yes	13,431	5.2% (703)	1.46	1.36 (1.24–1.49)	1.39 (1.30–1.50)	1.29 (1.20–1.40)
**Stroke**						
No	92,231	3.0% (2,740)	1.00	1.00	1.00	1.00
Yes	2,858	6.9% (197)	1.75	1.51 (1.29–1.76)	1.56 (1.38–1.77)	1.35 (1.17–1.56)
**Diabetes**						
No	85,510	2.8% (2,427)	1.00	1.00	1.00	1.00
Yes	9,579	5.3% (510)	1.60	1.31 (1.18–1.46)	1.37 (1.26–1.49)	1.24 (1.13–1.36)
**High blood pressure**						
No	60,644	2.6% (1,596)	1.00	1.00	1.00	1.00
Yes	34,445	3.9% (1,341)	1.26	1.20 (1.11–1.29)	1.28 (1.20–1.36)	1.14 (1.06–1.21)
**Any cardiovascular disease** ****						
No	77,928	2.6% (2,030)	1.00	1.00	1.00	1.00
Yes	17,161	5.3% (907)	1.56	1.43 (1.32–1.56)	1.45 (1.36–1.56)	1.34 (1.24–1.44)
**Vasectomy**						
No	70,023	3.3% (2,285)	1.00	1.00	1.00	1.00
Yes	25,066	2.6% (652)	0.96	1.04 (0.95–1.14)	0.99 (0.91–1.06)	1.05 (0.97–1.13)
**Erectile dysfunction**						
none	36,266	1.3% (471)	1.00	1.00	1.00	1.00
mild	22,118	2.3% (514)	1.60	1.52 (1.35–1.73)	1.47 (1.33–1.63)	1.48 (1.34–1.63)
moderate	14,878	4.6% (684)	2.95	2.53 (2.23–2.87)	2.35 (2.12–2.60)	2.24 (2.02–2.48)
severe	11,293	7.7% (873)	4.84	3.80 (3.32–4.34)	3.23 (2.90–3.59)	3.17 (2.84–3.53)
**Difficulty fathering children**						
No	84,104	3.0% (2,540)	1.00	1.00	1.00	1.00
Yes	6,897	3.4% (237)	1.24	1.26 (1.10–1.45)	1.22 (1.09–1.37)	1.31 (1.17–1.47)
**Requires help with daily tasks**						
No	87,443	2.7% (2,316)	1.00	1.00	1.00	1.00
Yes	4,009	11.1% (445)	3.93	2.94 (2.62–3.31)	2.61 (2.37–2.89)	2.52 (2.26–2.80)
**Physical functional limitation**						
None	32,163	1.3% (409)	1.00	1.00	1.00	1.00
Minor	27,927	2.0% (571)	1.40	1.37 (1.21–1.57)	1.39 (1.26–1.54)	1.38 (1.25–1.52)
Moderate	18,385	4.2% (770)	2.68	2.41 (2.12–2.74)	2.45 (2.22–2.71)	2.21 (1.99–2.45)
Severe limitation	8,499	9.6% (815)	6.42	5.17 (4.51–5.93)	4.48 (4.01–5.00)	4.23 (3.78–4.74)
**Psychological distress (K10 score**)						
Low (10–15)	70,370	2.0% (1,390)	1.00	1.00	1.00	1.00
Moderate (16–21)	13,088	5.0% (648)	3.00	2.75 (2.49–3.04)	2.42 (2.24–2.62)	2.43 (2.24–2.64)
High (22–50)	5,990	9.2% (553)	6.22	5.03 (4.50–5.62)	4.15 (3.77–4.56)	4.18 (3.79–4.60)
**Self-rated quality of life**						
Excellent	21,384	1.4% (306)	1.00	1.00	1.00	1.00
Very good	35,611	1.9% (684)	1.31	1.26 (1.10–1.45)	1.31 (1.18–1.45)	1.36 (1.22–1.51)
Good	25,405	3.9% (1,002)	2.61	2.31 (2.02–2.65)	2.18 (1.97–2.43)	2.13 (1.92–2.38)
Fair	7,465	7.8% (582)	5.31	4.29 (3.68–4.99)	3.98 (3.53–4.49)	3.79 (3.34–4.29)
Poor	1,512	14.5% (219)	11.48	8.69 (7.13–10.60)	7.04 (5.95–8.34)	6.51 (5.45–7.77)

*Excluding men with prostate cancer and/or previous prostate surgery.

**Total numbers in Table 4 do not include missing cases, but odds ratios and confidence intervals include the missing cases. Percentage missing: Erectile dysfunction  = 11.1%; Difficulty fathering children  = 4.3%; Requires help with daily tasks  = 3.8%; Physical functional limitation  = 8.5%; Psychological distress (K10 score)  = 5.9%; Self-rated quality of life  = 3.9%; other variables  = 0 missing.

***Adjusted for age, education, income, alcohol consumption, smoking, BMI and physical activity.

****Participants were categorised as having cardiovascular disease if they answered YES to any of the following ‘Has your doctor ever told you that you have heart disease/stroke/blood clot (thrombosis)?’

## Discussion

This large, population-based study of older Australian men adds to the growing body of evidence that while LUTS increase with age, a number of potentially modifiable risk factors including smoking, low physical activity and obesity are associated with severe LUTS. General ill-health and a range of comorbid conditions, some of which share these modifiable risk factors, were also associated with severe LUTS. The strong relationship observed between LUTS and physical functional limitation and general disability in men, regardless of its cause, has not, to our knowledge, been shown before. Consistent with previous research, we also found that nocturia was the most commonly reported symptom overall and that high storage symptom scores increased more with age than voiding symptoms [9]. This is one of the first studies with an adequate sample size to quantify risk factor associations with severe LUTS and to show relationships with age and in different sub-populations. It is also the largest population-based study of LUTS.

In the entire cohort severe LUTS were reported by 3% of men aged ≥45 years without previous prostate cancer and/or surgery, which is consistent with other studies [6], [14]. LUTS were particularly common in men reporting a previous diagnosis of an enlarged prostate (without previous prostate cancer or surgery), and men who had prostate cancer and/or a total or partial prostatectomy. These findings are consistent with various studies reporting quality of life outcomes in men post-prostatectomy, which show higher odds of urinary leakage among men who have surgery compared to no treatment or age-matched controls, but lower odds of weak stream or nocturia [22].

Evidence for a relationship between smoking and LUTS in previous studies has been mixed [6], [10], [23], [24], [25]. In our study, the odds of severe LUTS were 64% higher for current smokers and 31% higher for past smokers compared to never smokers. Alcohol consumption was associated with lower risks of both severe overall LUTS and voiding LUTS in our cohort, in contrast with a meta-analysis that found increased LUTS (but not BPH) with alcohol consumption [26].

Previous reports of increased LUTS with increasing body-mass-index [27] are consistent with our findings suggesting a stronger relationship with storage than with voiding symptoms. Increased levels of physical activity have consistently been associated with lower levels of LUTS [28], [29]. We also found an inverse relationship between physical activity and severe LUTS.

All of the comorbid conditions measured; heart disease, stroke, diabetes, high blood pressure and cardiovascular disease, were associated with higher odds of severe LUTS, even after adjusting for lifestyle risk factors. The mechanism for this is unclear however medications for these conditions may increase LUTS. We found an association between erectile dysfunction and severe LUTS, consistent with the evidence that several common pathophysiological mechanisms, risk factors and comorbidities are shared between both conditions [30]. These shared risk factors include diabetes, lipid disorders, metabolic syndrome and major cardiac diseases. Early diagnosis of either LUTS or erectile dysfunction provides an opportunity for clinicians to address and potentially modify risk factors that may reduce the risk of subsequent cardio vascular disease. Our finding of an increased risk of severe LUTS among men reporting difficultly fathering children has not, to our knowledge, been observed before and requires further exploration.

A major finding of the study is the strong and graded relationship of severe LUTS, and storage and voiding symptoms, to physical functional impairment and to general disability. The aetiology underlying these observations is not known, however it is likely to be in part related to the way in which morbidity influences LUTS. Furthermore, the strong relationship of disability to LUTS is likely to influence its relation to quality of life and psychological distress [31]. Nearly all LUTS are associated with some form of reduced quality of life or increased levels of anxiety and depression, with men who experience severe symptoms or multiple symptoms reporting the most impact on quality of life and mental health [1], [2], [32], [33], [34]; this is likely to reflect both the direct impact of LUTS and confounding due to disability and other health issues. Given the relationship of symptoms to quality of life, it is concerning that only a small proportion of men seek medical treatment for LUTS [35]. Reasons for this include a reluctance to discuss symptoms with a doctor, and the belief that LUTS are an inevitable part of ageing or are untreatable [35]. It is important that more is known about the aetiology of LUTS so that treatment and prevention can be optimised.

This study has a number of strengths, including a large sample size, sampling from the general population, use of a measure of LUTS that has been calibrated against the accepted international standard and the ascertainment of a large number of health-related factors using validated measures.

There are however limitations to the study. 1) Estimates of point prevalence in the 45 and Up Study should be interpreted with caution, given the low participation rate. However, the 21.9% prevalence of moderate-severe LUTS reported among the entire sample is very similar to previous Australian and international studies [5], [6], [7]. Moreover, empirical work from the 45 and Up Study shows that the effect size estimates from the Study are comparable to those from more representative studies and are generally not affected by response rates [16]. 2) Men with a prior history of prostate cancer or prostatectomy were excluded from the main analyses of this study. Therefore, our results represent the relationship of LUTS to lifestyle and health-related factors at a specific time point among men who have not had previous surgery and as a result may underestimate the burden of disease from severe LUTS in the population. 3) We lacked information on use of medications to treat LUTS, however this bias would tend to lead to conservative estimates of associations because users may have been misclassified as having lesser LUTS. 4) A cross-sectional analysis is unable to identify causal relationships and as such there is the possibility of reverse causality in a number of the associations described. For example, the direction of the relationship between LUTS and alcohol and LUTS and physical activity cannot be determined from these data.

Overall, the findings suggest that maintaining a healthy lifestyle is likely to relate to a lower risk of LUTS, even at older ages.

## Summary

As well as demonstrating strong relationships between disability and LUTS, this report confirms and quantifies a number of important risk factors for LUTS in men. In addition to low socioeconomic status and serious chronic disease, it identifies several modifiable risk factors that are associated with severe LUTS, including smoking, lack of physical activity and obesity. These factors, if modified, may reduce the risk of LUTS, as well as a number of other chronic diseases, in men.
